# Genetic Liability to Attention-Deficit/Hyperactivity Disorder and Economic Outcomes

**DOI:** 10.1192/j.eurpsy.2026.10239

**Published:** 2026-07-31

**Authors:** A. Hazak, J. Liuhanen, K. Kantojärvi, S. Sulkava, T. Jääskeläinen, V. Salomaa, S. Koskinen, M. Perola, T. Paunio

**Affiliations:** 1 Department of Public Health, Finnish Institute for Health and Welfare; 2 Research Programs Unit, Faculty of Medicine, University of Helsinki, Helsinki; 3 Department of Finance, Aalto University, Espoo, Finland; 4 Department of Economics and Finance, Tallinn University of Technology, Tallinn, Estonia; 5 Department of Psychiatry / SleepWell Research Program, University of Helsinki and HUS Helsinki University Hospital; 6 Department of Clinical Genetics, HUS Helsinki University Hospital, Helsinki; 7 Department of Internal Medicine, University of Turku, Turku; 8 Research Program for Clinical and Molecular Metabolism, University of Helsinki, Helsinki, Finland

## Abstract

**Introduction:**

Attention-Deficit/Hyperactivity Disorder (ADHD) is characterised by cognitive and behavioural tendencies that extend beyond clinical symptoms and can shape social and economic participation. Polygenic scores (PGS) increasingly enable the study of genetic predispositions in population-based settings, offering insight into how inherited liability—regardless of clinical manifestation—may influence life-course outcomes.

**Objectives:**

This study examined how genetic liability to ADHD relates to a range of economic outcomes in the general population.

**Methods:**

We analysed six Finnish population-based cohorts collected between 1992 and 2017 (N ≈ 20,000; ages 25–64), linking registry, survey and genetic data. PGS for ADHD was derived from large-scale genome-wide association studies (Demontis *et al.* Nature Genetics 2019; 51 63–75) and applied in regression models of educational and labour market outcomes. Models adjusted for demographic and genetic ancestry covariates.

**Results:**

Higher PGS for ADHD were associated with less favourable outcomes across multiple domains of economic performance. Individuals with elevated genetic liability were disadvantaged in both educational attainment and labour market participation. Additional economic disadvantages were largely attributable to earlier sorting into educational and occupational pathways. Moreover, the study offers novel evidence that higher ADHD-PGS is linked not only to objective markers of economic status but also to subjective economic wellbeing.

**Conclusions:**

Genetic predisposition to ADHD is linked to economic disadvantage through multiple pathways across the life course. These findings highlight that ADHD-related genetic liability relates to socio-economic inequality beyond the clinically diagnosed population.

**Disclosure of Interest:**

A. Hazak: None Declared, J. Liuhanen: None Declared, K. Kantojärvi: None Declared, S. Sulkava: None Declared, T. Jääskeläinen: None Declared, V. Salomaa: None Declared, S. Koskinen: None Declared, M. Perola: None Declared, T. Paunio Consultant of: Idorsia Pharmaceuticals and Biogen (unrelated to the present work)

